# Mediastinal desmoplastic small round cell tumor

**DOI:** 10.1097/MD.0000000000022921

**Published:** 2020-10-30

**Authors:** Dacheng Jin, Meng Chen, Bing Wang, Yunjiu Gou

**Affiliations:** a The first department of thoracic Surgery, Gansu Provincial Hospital; b Department of Clinical Medicine, Gansu University of Traditional Chinese Medicine, Lanzhou, PR China.

**Keywords:** chemotherapy, desmoplastic small round cell tumor, mediastinum

## Abstract

**Rationale:**

Desmoplastic small round cell tumor (DSRCT) is a rare distinct tumor with a high-grade malignancy.

**Patient concerns:**

A 51-year-old male visited a local hospital in April 2016 complaining of shortness of breath, chest tightness and pain, and exhibited significant swelling in both sides of the chest.

**Diagnoses:**

CT demonstrated thoracic symmetry and no abnormalities were observed in the soft tissues of the ribs and the chest wall. A general observation of CT-guided puncture biopsy revealed 2 stripes of gray and grayish-white puncture tissues of 0.5 and 1 cm in length, respectively, and 0.1 cm in diameter. These results preliminarily suggested a (mediastinum) malignant small round cell tumor.

**Intervention:**

Given the progression of the disease, the chemotherapy regimen, consisting of ifosfamide and etoposide, was altered during the course and radiotherapy (total of 70 Gy of mediastinal Y field radiation) was conducted.

**Outcomes:**

The patient and his family declined further treatment. Through follow-up, the total survival period was determined as 17 months.

**Lessons:**

DSRCT is a rare interstitial malignant tumor. Effective cytoreduction combined with comprehensive therapies could achieve partial remission or prolong the survival of patients.

## 1 Introduction

Desmoplastic small round cell tumor (DSRCT) is a rarely-seen distinct tumor with high-grade malignancy. There have been less than 400 cases reported worldwide, with 95% of cases found in the abdominal cavity.^[1]^ Common sites of lesion include the posterior peritoneum, pelvic cavity, omentum, and mesentery. The tumor can also be occasionally found in the testicles, ovaries, liver, tongue root, and nervous system, but rarely found in the chest. There have been a few cases of pulmonary and pleural DSRCT^[1–6]^ but cases of primary DSRCT in the mediastinum remain scarce. A patient with mediastinal DSRCT had been admitted to our hospital and the case is reported as follows.

## 2 Case profile

**Figure 1:**
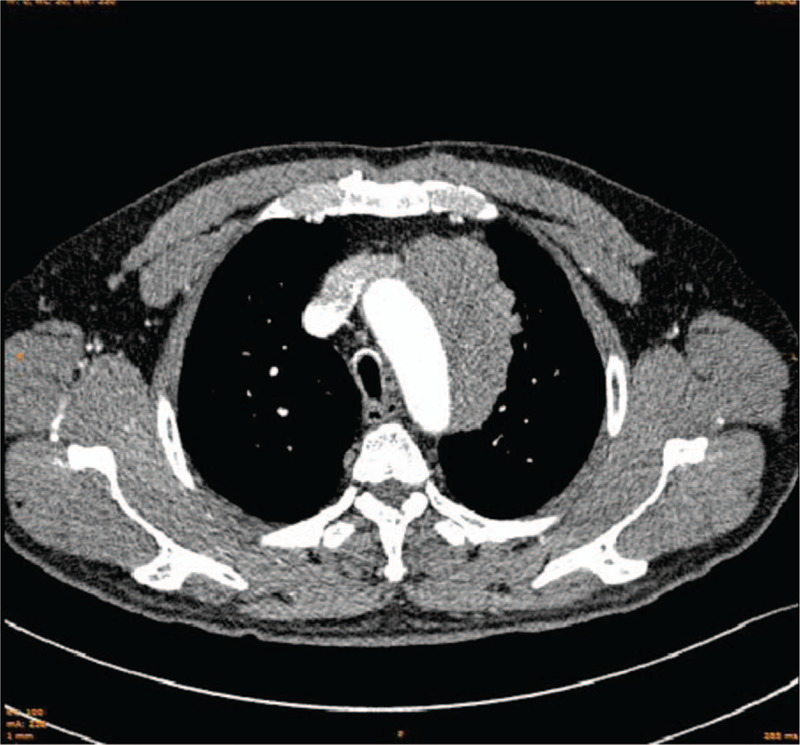
Enhanced CT showed malignant tumor in the middle and upper mediastinum on the left side.

**Figure 2:**
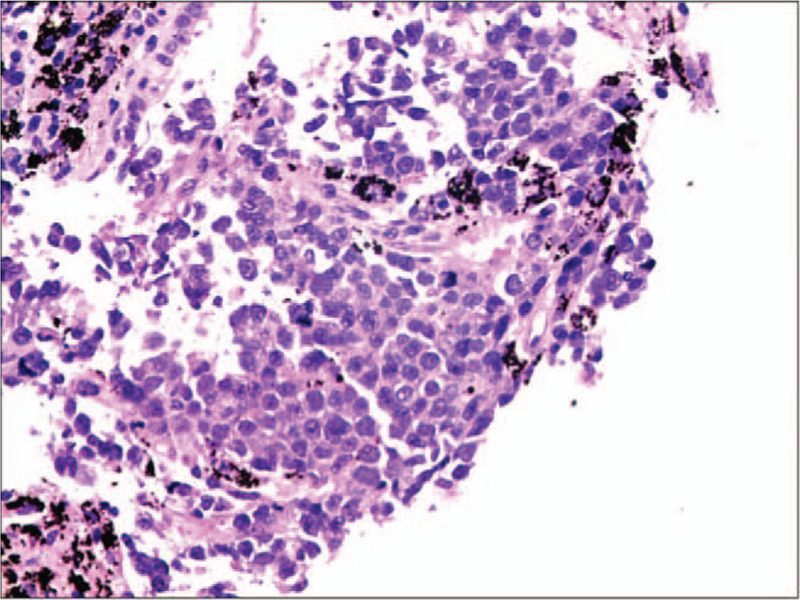
Histopathological results suggested that the cells were small and round, with cell nest surrounded by fibrous tissues (HE, ×400). Immunohistochemistry suggested that desmin, PCK, vimentin, CD99, EMA scattered lesions were positive, while MyoD1, WT-1, LCA, NSE, CgA, DOG1, SMA, CD34, Bcl-2, myoglobin, myogenin, S-100, and BCL-2 were negative.

The 51-year-old male patient visited a local hospital for complaint of chest tightness, shortness of breath, and chest pain in April 2016, with significant swelling pain in both sides of the chest. CT showed thoracic symmetry and no abnormalities in the soft tissues of the ribs and chest wall. The pulmonary window showed increased, thickened, and disorderly texture of the lungs. The mediastinal window showed fan-shaped intensity of soft tissue on the left anterior side of the large blood vessel. The maximum level was about 8.2∗3.4 cm in size, with lobulated boundary. The mediastinum was not dislocated. Increased heart shadow and left pleural hypertrophy and calcification were found. After symptomatic treatment (with specific drugs usage and dosage unknown) provided at the local hospital, the patient did not experience improvements of symptoms. During the course of the disease, there was no hemoptysis, blood in the sputum, hoarseness, nausea, vomiting, or abdominal pain. The patient visited our hospital on August 13, 2016 for further diagnosis and treatment. After admission, CT showed mediastinal mass in the middle and upper parts of the left side, which was considered as a lymphoma, and a puncture biopsy was suggested for verification. Left upper lobe fibrous cords and calcifications were found. The left hilar and the mediastinum were found with multiple lymph node enlargements, and a little effusion was found in the pericardium. Thickening and calcification were detected in the right pleura (Fig. 1). CT-guided puncture biopsy showed that, in general observation, there were 2 stripes of gray and grayish white puncture tissues with 0.5 and 1 cm in length, respectively, and 0.1 cm in diameter. The results preliminarily suggested (mediastinum) malignant small round cell tumor (Fig. 2). Immunohistochemistry suggested desmin, PCK, vimentin, CD99, and EMA scattered lesions were positive, while MyoD1, WT-1, LCA, NSE, CgA, DOG1, SMA, CD34, Bcl-2, myoglobin, myogenin, S-100, and BCL-2 were negative. Given the high-grade malignancy of the tumor, as well as invasion of the large blood vessel, no surgery was performed. Treatment was provided with cyclophosphamide combined with doxorubicin and vincristine chemotherapy for 4 cycles. On March 25, 2017, the patient was admitted to the hospital again. CT re-examination showed:

1. The brain parenchyma had multiple round shadows of slightly low intensities, with peripheral edema; according to medical history, possibility of metastases was considered.
2. A malignant space-occupying lesion was found in the middle and upper mediastinum on the left side, with its scope slightly greater than before.
3. Thickening and calcification of the left pleura were detected.
4. Multiple fibrous cords and calcifications in the left lung as before;
5. The left hilar and the mediastinum were found with multiple lymph node enlargements.
6. Left adrenal nodules were considered as metastases.

Use ifosfamide (9 g/m^2^) + etoposide(500 g/m^2^) every half month. It lasted 3 cycles. Subsequently, cyclophosphamide(200 g/m^2^) + adriamycin(75 g/m^2^) + vincristine(1.4 g/m^2^) were used for maintenance treatment for 4 cycles. And radiotherapy (a total of 70Gy of mediastinal Y field radiation) was conducted. No significant adverse reactions were observed during chemotherapy and radiotherapy. However, no improvement was achieved, and the patient and his family gave up further treatment. Through follow-up, the total survival period was 17 months.

## 3 Discussion

### 3.1 Clinical manifestations of DSRCT

DSRCT is a rare highly malignant tumor commonly found in the abdomen, pelvic cavity, and extra-abdominal organs. It occurs mainly in adolescents, with a male to female ratio being approximately 4:1.^[6–8]^ Currently, most cases of DSRCT have been reported to be found in the peritoneum, ovary, liver, kidney, pancreas, bone, skull, head, and neck, but there are also a few cases of pulmonary and pleural DSRCT reported.^[9–13]^ The disease does not present specific clinical signs and symptoms. Patients may suffer from abdominal distension, abdominal pain, abdominal mass, ascites, urinary tract irritation, hepatomegaly, constipation, and intestinal obstruction.^[14–16]^ DSRCT primarily occurred in the chest is extremely rare. Patients may have symptoms of coughing and difficulty in breathing. Patients with pleural involvement are often complicated by chest pain and tightness. The pleural DSRCT progresses rapidly and can be metastasized to the abdominal and pelvic cavities in the advanced stage, with poor prognosis.^[17,18]^ The male patient in this case visited the hospital complaining of chest tightness, shortness of breath, and swelling pain in chest.

### 3.2 Pathological and imaging features of DSRCT

Histologically, DSRCT is difficult to be distinguished from neuroblastoma, rhabdomyosarcoma, Ewings sarcoma, primitive neuroectodermal tumor, and synovial sarcoma, all of which are collectively referred to as the malignant small round cell tumors. However, DSRCT has its unique immunohistochemical and chromosomal ectopic results. At present, the histological features of DSRCT are summarized as well-defined nested, island-like, or stripe-like distribution of small round cells surrounded by a large number of fibrous interstitials.^[19]^ Hemorrhagic and necrotic areas can be seen inside the tumor, and there are stromal tumor vessels with obvious hypertrophy in the hemorrhagic area. Immunohistochemical results typically indicate multi-directional differentiation of tumor cells. Epithelial-derived cytokeratin, epithelial membrane antigen, interstitial marker vimentin, myogenic marker intermuscular line protein, nerve cell adhesion molecule, and neuron-specific enolase all show positive results. In this case, immunohistochemical results indicated that desmin, PCK, vimentin, CD99, and EMA scattered lesions were positive, which was basically consistent with the features of DSRCT from the pathological perspective. Diverse differentiation is one of the significant features of DSRCT. Almost all the cases of DSRCT may suggest epithelial, interstitial, and neuronal marker activities, which is basically similar to that of pleural and other cases of DSRCT. Some studies^[20]^ have confirmed that patients with DSRCT all have t (11:22) (p13; p12) chromosomal translocations, leading to the expression of the fusion gene EWS-WT1. There are also studies^[21]^ suggesting that EWS-WT1 fusion protein can activate the transcription of insulin-like growth factor-1. Therefore, the presence of fusion protein may be the essence of DSRCT cell proliferation.

Characteristic features are absent in DSRCT's imaging, and examinations usually include B-ultrasound, CT, and MRI. CT of abdominal lesions usually reveals single or multiple soft tissue masses. When there is a necrotic area in the tumor, it is usually accompanied by flakes of low intensities. Enhanced CT presents mild uneven enhancement. Since the cells of DSRCT are closely packed and rich in fibrous tissue, some patients may show mild enhancement in the arterial phase and no prolonged enhancement or clearance during the venous and extended phases. MRI usually presents equal or low signal of T1 weighted phase heterogeneity, high signal of T2 weighted phase unevenness, and uneven mild enhancement in enhanced scanning.^[22]^ There have been few reports on the pleural DSRCT, and the imaging features lack specificity. Li Gang^[4]^ reported 5 cases of pleural DSRCT, including 2 cases of primary occurrence in the pleura, 1 in the mediastinum, and 2 in the lungs. It is generally manifested as diffuse growth along the pleura. DSRCT primarily occurred in the pleura often involves the pericardium, lungs, and mediastinum, so it is difficult to distinguish it from advanced lung cancer and mediastinal tumor. Zuo Dingxiang^[11]^ reported a case of pulmonary DSRCT, in which the patient had been preoperatively diagnosed with CT as silicosis and infection. This case was later confirmed by postoperative examination and comprehensive analysis. In summary, the imaging of DSRCT lacks specificity, making it difficult to be distinguished from other diseases. The diagnosis needs to be confirmed with surgical specimen or biopsy.

### 3.3 Differential diagnosis of DSRCT

DSRCT is a type of malignant small round cell tumors and therefore needs to be distinguished from neuroblastoma, rhabdomyosarcoma, Ewings sarcoma, primitive neuroectodermal tumor (PNET) and synovial sarcoma.

1. Metastatic small cell carcinoma: Although DSRCT cells present nested, island-like, or stripe-like distribution, they simultaneously express actin, desmin, and vimentin. For this reason, they can be easily identified and distinguished from metastatic small cell carcinoma.
2. PNET: DSRCT is similar to PNET, as both their forms are small cells encircling like chrysanthemum, and both have positive expression of CD99 and NSE. However, DSRCT shows the expression of EMA and desmin, with a large amount of interstitial hyperplasia of fibrous tissues.
3. Small cell malignant mesothelioma

They are usually arranged in a lamellar-like structure, while both MC and calretinin are negative in DSRCTs immunohistochemistry, simultaneously expressing markers of nerve, muscle, and epithelial tissues. DSRCT should also be distinguished from other diseases such as malignant lymphoma and small cell lung cancer. Pulmonary small cell malignant tumors generally present no significant fibrous tissue proliferation, and the nuclear nucleoli of cancer cells are unclear. Although they express EMA and NSE, the expressions of MyoD1, WT1, and myogenin are absent. Lymphocytic lymphoma in malignant lymphoma is commonly seen in the mediastinum, but its cells are generally diffused in a sheet form, often involving bone marrow and peripheral blood, so they can be distinguished.

### 3.4 Treatment of DSRCT

There is currently no standard therapeutic regimen for DSRCT. According to a review of previous literature, treatment methods mainly included surgery, chemotherapy, radiotherapy, and targeted drug therapy. Studies have reported that effective cytoreduction can reduce tumor burden. Although it is impossible to achieve complete excision, radical surgical resection can prolong the survival of patients.^[23,24]^ Biswas et al^[24]^ concluded that effective surgical resection could extend patients survival, but the approach was usually limited to early non-metastatic DSRCT. Lal et al^[25]^ compared the 3-year survival of the surgery group and the non-surgery group, and the results showed that the 3-year survival was 58% in the surgery group, while there was no survivor in the non-surgery group. This suggested that effective cytoreduction and R0 resection would be beneficial to early-stage patients with no distant metastasis.^[26,27]^ Some researchers^[28,29]^ have also found that a combination of neoadjuvant chemotherapy and surgery not only brings patients an opportunity of radical surgery, it is also of certain significance for patients who can tolerate secondary surgery by adopting high-intensity radiochemotherapy combined with bone marrow transplantation. Li Gang et al^[4]^ reviewed 5 patients with DSRCT primarily occurred in the chest. It was found that 2 patients at the advanced stage of disease with distant metastases had a survival of 25 and 16 months, respectively. Partial remission occurred in both patients during the period, indicating that radiochemotherapy was still effective for advanced DSRCT in patients who cannot undergo surgery. Two patients who merely underwent surgery still survived during a follow-up of 2 and 10 months, indicating that effective cytoreduction had a certain positive impact on prolonging survival. The effects of chemotherapy regimens for early DSRCT have not been desirable, and the most representative P6 program has been reported repeatedly and applied in clinical practice. Kushner et al^[30]^ performed P6 (the first to third cycles and the sixth cycle with cyclophosphamide + doxorubicin + vincristine; the fourth, fifth, and seventh cycles with ifosfamide + etoposide) chemotherapy and surgical treatment on 12 patients. It was found that P6 chemotherapy combined with cytoreduction could achieve partial remission in clinical practice. However, due to the high dose of chemotherapy used by Kushner et al^[30]^, the patients survival was relatively short. For this reason, it is recommended that chemotherapy should be applied with appropriate dose. In this case, after the patients were diagnosed with DSRCT, chemotherapy using cyclophosphamide combined with doxorubicin and vincristine was provided for 4 cycles, during which partial remission was achieved. Unfortunately, due to the rapid progression of the disease complicated by distant metastasis, the best timing for surgery was missed, hence the patients survival only lasted for 21 months. The application of targeted drugs has been rarely reported. Currently, the use of targeted drugs such as sunitinib, imatinib, sirolimus combined with IGFR monoclonal antibody, and trastuzumab has been proven beneficial to patients. However, such treatment is more selective for patients, therefore the application of targeted therapy for DSRCT remains in the preliminary stage at present.

In summary, DSRCT is a rare interstitial malignant tumor. In this case, the tumor was found in the mediastinum, suggesting that the tumor might not have a specific site of onset. Moreover, no specificity was shown in imaging findings, and the diagnosis mostly relies on histological and genetic testing results. The disease is characterized by high invasiveness, rapid progression, and high recurrence. Effective cytoreduction combined with comprehensive therapies could achieve partial remission or prolong the survival of patients.

## Author contributions

**Conceptualization:** Yunjiu Gou.

**Funding acquisition:** Yunjiu Gou.

**Methodology:** Dacheng Jin.

**Resources:** Dacheng Jin.

**Software:** Dacheng Jin, Meng Chen.

**Supervision:** Meng Chen.

**Validation:** Bing Wang.

**Writing – original draft:** Dacheng Jin.

**Writing – review & editing:** Dacheng Jin.
